# Public health impact of antihypertensive medication use on arterial blood pressure: A pooled cross-sectional analysis of population health surveys

**DOI:** 10.1371/journal.pone.0290344

**Published:** 2023-08-21

**Authors:** Diego Montano

**Affiliations:** Department of Population-Based Medicine, University of Tübingen, Tübingen, Germany; Houston Methodist Academic Institute, UNITED STATES

## Abstract

The early initiation of antihypertensive drug therapy is conceived as one of the most important public health interventions addressing cardiovascular risk in the population. However, the actual contribution of this public health intervention to reduce blood pressure (BP) at the population level is largely unknown. Hence, the aim of the present investigation is to estimate the potential public health effects of the use of antihypertensive medication on BP in the population aged 16 and older. Data from three population health surveys periodically conducted in the United States, England, and Scotland are analysed (*N* = 362,275). The secular trends of BP measurements and the potential public health impact of the use of antihypertensive medications on BP over time are analysed in a series of linear mixed models. Between 1992 and 2019, a secular trend of decreasing systolic and diastolic BP occurred (−16.24 99% CI [−16.80; −15.68] and −3.08 99% CI [−3.36; −2.80] mmHg, respectively). The potential public health impact of the use of antihypertensive medications in the period 1992–2019 on systolic BP was estimated to lie between −8.56 99% CI [−8.34; −8.77] and −8.68 99% CI [−8.33; −9.03] mmHg. Average reduction of diastolic BP was in the range of −5.56 99% CI [−5.71; −5.42] and −6.55 99% CI [−6.78; −6.32] mmHg. The observed changes in the distribution of BP measurements over time were found to be more strongly related to secular trends affecting the whole populations, rather than to increases in the proportion of individuals taking antihypertensive medications.

## Introduction

The effectiveness of antihypertensive medications in reducing the incidence of major cardiovascular events has been well documented in several meta-analytic studies of randomised-controlled clinical studies [1–3]. The most common antihypertensive medications comprise diuretics, angiotensin-converting enzyme inhibitors (ACE), angiotensin-receptor blockers (ARB), beta blockers, and calcium-channel blockers. Despite the fact that each medication class differs regarding its particular mode of action, previous research findings have suggested that the effects of different antihypertensives on blood pressure (BP) are rather similar [4, 5]. Notwithstanding some disagreements regarding the recommendations for the initiation of antihypertensive drug therapy [6], there is rather a general consensus in the major cardiology guidelines for the management of hypertension, e.g., ESC/ESH Guidelines, that for individuals with high cardiovascular risk, antihypertensive drug treatment should be considered as soon as blood pressure (BP) values exceed the high-normal threshold, defined at 130/85 mmHg [7]. From a public health perspective, the early initiation of antihypertensive drug therapy is conceived as one of the most important medical interventions to reduce the burden of cardiovascular risk and disease in the population [8]. In fact, the Joint National Committee on Prevention, Detection, Evaluation, and Treatment of High Blood Pressure in the United States has reclassified BP levels between 120–129/80–84 mmHg as a form or pre-hypertension which, depending on the individual’s risk for cardiovascular disease, may qualify for the initiation of antihypertensive drug therapy [9].

As a consequence, since the late 1990s, hypertension awareness and treatment in the adult population have increased in several high-income countries, including the United Kingdom, Germany, Japan, and the United States, where currently about 50% to 80% of the adult population diagnosed with arterial hypertension report taking one or more antihypertensive medications [10]. The prevalence of hypertension in the adult population in high-income countries varies between 30% and 50% [11, 12], with estimated prevalence rates among persons 70 years and older increasing to about 70% [10]. On the other hand, however, BP control, especially systolic BP, is difficult to achieve in practice and clinical settings, even for uncomplicated hypertension cases for which the treatment goal threshold is 140/90 mmHg [13]. For instance, it has been reported that in the U.S. adult population, only about 48% of hypertensive adults achieved the BP control threshold [14].

Furthermore, the estimation of the potential impact of antihypertensive medications on population health is challenging due to the effect of different factors that may influence the distribution of BP in the adult population. For instance, non-adherence, improper antihypertensive regimes, or the so-called resistant hypertension—defined as BP elevated above goal despite the use of different classes of antihypertensives, usually a calcium-channel blocker, a renin-angiotensin blocker, and a diuretic [15]—imply that BP control in some proportion of the population is not likely to be achieved [16]. In addition, given the complex physiological mechanisms responsible for BP regulation, the distribution of BP measurements in the population can also be influenced by changes in the distribution of several factors directly or indirectly affecting BP measurements. For instance, in a seminal investigation of the NCD Risk Factor Collaboration (NCD-RisC) with data from 1018 population-based BP measurement studies, there was evidence that average BP levels have been driven mostly by secular trends, rather than by treatment of individuals with high BP levels [17]. However, it is still largely unknown to what extent antihypertensive drug therapy has contributed to a reduction of BP measurements at the population level in comparison to secular changes related to various factors such as nutrition, body weight, or physical activity, which may ultimately account for the largest effect on average BP levels [17].

Given the increasing public health relevance of antihypertensive medications, the major aim of the present investigation is to estimate the potential public health impact of the use of antihypertensive medications on the distribution of BP measurements at the population level. This is important, among other reasons, in order to assess the impact of antihypertensive therapy under routine clinical practice and to quantify the extent to which additional reductions in BP at the population level could be attained. Accordingly, the analysis will focus on how BP measurements have changed over time within sub-populations of individuals taking antihypertensive medication in comparison to the general population over a period of about 30 years in three large representative health surveys conducted periodically in England, Scotland, and the United States. The present study contributes to previous research in three ways: (i) it provides estimates of secular trends of BP measurements and the potential treatment effects of the use antihypertensive medication at the population level; (ii) temporal changes in body mass index (BMI), smoking behaviour, and demographic structure of the population and their impact on BP measurements are taken explicitly into account; and (iii) the variation of BP measurements within and across countries is considered in relation to the proportion of individuals taking one or more antihypertensive medications. Even though the previous study of NCD-RisC has suggested that secular trends in the population have been the driving forces determining the changes in the average BP levels [17], to the knowledge of the author, a detailed analysis of individual data on the associations between the use of specific antihypertensive medications in the population and the changing distribution of BP measurements over time in different populations has not been performed yet.

## Materials and methods

### Data

The investigation of the main question in the present study will be based on data from three population health surveys conducted periodically in the United States (U.S.), England, and Scotland. From a very general perspective, the surveys are random probability samples of the population in each country and consist of extensive questionnaires and standardised examination data on magnitudes such as BP, anthropometric measurements, blood and urine analytes, medical conditions, prescription medications, and socio-economic indicators. For all surveys, respondents age 16 and older with valid measurements were included in the analyses. The Health Survey for England (HSE) is a stratified random sample of private English households [18]. In the present study, HSE data from 1992 to 2019 comprising 28 survey years were considered for the analyses. The National Health and Nutrition Examination Survey (NHANES) is a periodic examination and health survey of the civilian, non-institutionalised population of the United States [19]. The present study includes 11 NHANES survey years conducted between 1994 and 2018. The third survey is the Scottish Health Survey (SHS), a random sample of private households in Scotland with a similar scope and structure as the HSE [20]. In the present analyses, SHS data from 14 survey years collected between 1995 and 2019 were considered.

### Variable harmonisation

In order to ensure the comparability of measurements within and between surveys and to pool the data from the three surveys, the time series of the variables of interest were harmonised by taking into consideration the data collection protocols used in the different survey waves. The complete list of original variables used for harmonisation in each survey and year is provided in the S1 Appendix.

*Demographic and anthropometric Information* correspond to sex and age of survey participants at the time of interview. Age is categorised within and across surveys as an ordinal variable including four age bands: 16–29, 30–49, 50–64 and 65 years and older. Anthropometric measures are weight in kilograms and height in metres, which were used to calculate the body mass index (BMI).

*Use of antihypertensives*. Information on the use of the four most commonly prescribed antihypertensive medications was available in the questionnaire data of each survey, namely: (1) diuretics (ATC code C03), (2) beta blockers (ATC code C07), (3) calcium-channel blockers (ATC code C08), and (4) angiotensin-converting enzyme (ACE) inhibitors (ATC code C09). In NHANES, except for the 1994 wave, prescription medications reported by respondents are coded in a 3-level nested category system (Multum Lexikon) that assigns a therapeutic classification to each reported medication and each ingredient of the medication. In 1994, prescription medications were coded by using a database of the Product Information Branch of the Food and Drug Administration (primary mode of action of up to 14 medications), and for subsequent NHANES survey years, the second level of up to four medications were used to identify the four major antihypertensive drug classes [21]. In HSE and SHS, the use of antihypertensive medication is provided as single dichotomous variables that are derived from the individual medication codes assigned during the standardised interviews (e.g., [22, 23]). The consistency of the self-reported information on antihypertensive drug intake was checked against the information supplied by respondents on whether they were currently taking any medication. In addition, by considering that hypertension therapy usually involves taking more than one antihypertensive medication, a new variable with four categories was derived that indicates whether the person does not take any medication at all (“no medication”), takes one antihypertensive drug (“monotherapy”), or takes more than one (“combined therapy”). The fourth category, “other medications”, includes information on any other type of medications taken by the survey participants. Depending on the data collection protocols of each survey, this category may include a vast array of different medications such as antidiabetics, systemic hormonal preparations, stomatological preparations, anti-infectives, or analgesics. Hence, the category “other medications” controls for potential effects of other medications on BP measurements.

*Blood pressure measurements*. In each survey, the measurement of systolic and diastolic BP follows a standardised protocol with specific instructions for physicians in NHANES or study nurses in HSE and SHS (see e.g., [24, 25]). BP measurements are usually taken in the right arm while respondents sit up straight in a comfortable position. Respondents are asked not to eat, smoke, drink alcohol, or participate in vigorous activities 30 min before BP measurement and to remain quiet for about 5 min. In the HSE and SHS samples, BP measurements are collected by automatic blood pressure devices (e.g., DINAMAP and OMRON HEM PB) and reported as the average of the second and third measurements (or average of all measurements in SHS) [20, 22, 24]. The protocol for BP measurement in NHANES consists of up to four sphygmomanometer readings whose procedure has been described in detail elsewhere [25]. According to the NHANES protocol [25], the BP average of the last two readings is calculated if available. If only one reading was obtained, that reading is used as the average. Readings of diastolic BP less than 30 mmHg were considered missing.

*Sampling design*. In order to account for the fact that all surveys are based on some form of cluster and/or stratified sampling, the sampling strata of the surveys were included in the analyses. For the HSE and SHS surveys, the regional units in Scotland and England, and for the NHANES samples, the masked variance pseudo-stratum, were utilised to estimate the cluster sampling variance in the different survey waves.

### Statistical analysis

The time trends of systolic and diastolic BP measurements for all datasets considered in the present study are analysed by estimating unadjusted and adjusted linear-mixed regression models [26]. The random-effects are calculated at the level of the harmonised sampling strata of each survey year. The following models are specified:

$$Y_{sys,dia}=\{\begin{array}{l} \beta_{0}+\beta_{1}X_{a}+\beta_{2}(X_{a}\times X_{year})+b_{1}\cdot I_{strata}+\epsilon \\ \beta_{0}+\beta_{1}X_{a}+\beta_{2}(X_{a}\times X_{year})+\beta_{3}X_{adj}+b_{1}\cdot I_{strata}+\epsilon \end{array}$$ (1)

where *Y*_*sys*,*dia*_ is the dependent variable, i.e., either systolic or diastolic BP; *X*_*a*_ the variable capturing the use of antihypertensive drugs with four categories: no medication, monotherapy, combined therapy, and other medications; *X*_*year*_ the survey year; and **X**_*adj*_ a matrix including the individual characteristics of participants (sex, age, smoking behaviour. and BMI) and, for the pooled analyses, dummy variables for the surveys. The matrix **I**_*strata*_ is an identity matrix used to calculate the random intercepts *b*_1_, which vary according to the sampling strata of each survey year. The coefficients *β*_0_, *β*_1_, *β*_2_, *β*_3_ correspond to the intercept and fixed-effects estimates and, finally, *ϵ* is a vector with residual variation. The coefficient *β*_1_ expresses the overall BP differences between sub-populations taking antihypertensive medications, other medications, or no medications, whereas *β*_2_ indicates the potential public health impact of the use of antihypertensives (i.e., time vs. treatment interaction effects). The secular trends of BP measurements are captured by the main effects corresponding to the survey year. The strata-specific variation should take into account changes in the data collection protocols over time and survey-specific characteristics that may have had an impact on overall BP variance.

It has to be emphasised that the statistical analysis of the present study does not pursue the estimation of the treatment effects of antihypertensive medications, but the extent to which they have contributed to the reduction of average BP measurements at the population level. As stated in the introduction section, antihypertensives have already been found to be effective in reducing BP in large randomised-controlled prospective clinical studies. Hence, the estimates that are of relevance for this study are the interaction effects *β*_2_, i.e., the rate of reduction of BP within sub-populations in comparison to non-treated individuals. The coefficient *β*_1_ adjusts the magnitude of the interaction effects for the fact that antihypertensive drug therapy is indicated precisely for those individuals having high BP measurements. Therefore, the regression estimates of the public health impact of antihypertensive medications over time are valid marginal effect size estimates of the differences between untreated and treated individuals after taking into account secular (unknown) influences affecting whole populations and the non-ignorable assignment to treatment (i.e., *β*_1_). In addition, the robustness of the estimates is enhanced by conditioning on a set of relevant individual characteristics (age, BMI, sex) and time, regional and country-specific variations that may directly influence the assignment to antihypertensive treatment. Due to space limitations, the results of the unadjusted regression models are reported in the S1 Appendix. The reported confidence intervals were estimated at the 99% level to reduce the probability of false positives for small effects in large samples [27]. All statistical analyses were performed in the statistical environment R, specifically with estimation routines for linear mixed models implemented in the package lme4.

## Ethics statement

The present study is conducted with secondary data which have been already collected by the local health authorities of the countries included in the analysis. The surveys fulfil ethical guidelines and regulations, including written consent to participate. Further details on ethical issues are available for the general public in the websites of the corresponding surveys at https://ukdataservice.ac.uk/ and https://www.cdc.gov/nchs/nhanes/.

## Results

### Descriptive statistics

A total of 362,275 observations of respondents with valid systolic and diastolic BP measurements were included in the pooled dataset (Table 1). The HSE and SHS samples included more smokers and a slightly younger population than the NHANES samples. In comparison to the HSE and SHS, the NHANES samples have a larger proportion of individuals either taking medications in general or being under combined antihypertensive therapy, in particular. Across surveys, calcium-channel blockers are less frequently prescribed than diuretics, beta blockers, and ACE inhibitors. On the other hand, whereas systolic and diastolic BP levels are frequently higher in HSE and SHS than in NHANES, the proportion of individuals with a BMI of more than 35 is largest in NHANES.

**Table 1:** Descriptive statistics of the surveys for all data collection years combined. Proportions (%) and frequencies (in parentheses) for categorical variables and means and standard deviations (in parentheses) for systolic and diastolic blood pressure.

Variable	Category	NHANES	HSE	SHS
Sex	Female	53 (78,954)	55 (103,818)	56 (13,593)
	Male	47 (69,586)	45 (85,734)	44 (10,590)
Age	16–29 years	16 (23,886)	18 (33,475)	15 (3556)
	30–49 years	23 (33,620)	35 (65,980)	35 (8458)
	50–64 years	26 (38,089)	23 (44,216)	27 (6608)
	+65 years	36 (52,945)	24 (45,881)	23 (5561)
Body mass index	10–25	28 (41,298)	40 (71,289)	35 (7813)
	26–30	32 (45,941)	38 (68,324)	38 (8585)
	31–35	21 (31,163)	16 (27,682)	19 (4159)
	+35	19 (27,105)	6 (11,180)	8 (1849)
At least 1 antihypertensive medication?	no	57 (85,065)	81 (154,040)	80 (19,301)
	yes	43 (63,030)	19 (35,422)	20 (4824)
Antihypertensive drug use	No medication	25 (37,005)	52 (99,005)	47 (11,340)
	Monotherapy	22 (32,868)	11 (20,098)	11 (2565)
	Combined therapy	20 (30,162)	8 (15,324)	9 (2259)
	Other medications	32 (48,060)	29 (55,035)	33 (7961)
Currently smoking	No	80 (111,578)	69 (84,740)	59 (8545)
	Yes	20 (27,904)	31 (38,929)	41 (6042)
Diuretics	No	77 (93,105)	87 (112,311)	81 (9048)
	Yes	23 (27,797)	13 (16,347)	19 (2086)
Beta blockers	No	74 (90,005)	90 (116,259)	84 (9336)
	Yes	26 (30,897)	10 (12,213)	16 (1796)
ACE inhibitors	No	77 (93,093)	88 (114,025)	79 (8417)
	Yes	23 (27,809)	12 (15,139)	21 (2296)
Calcium-channel blockers	No	85 (103,134)	91 (117,219)	85 (9099)
	Yes	15 (17,768)	9 (11,355)	15 (1621)
Systolic blood pressure		126.57 (20.41)	131.72 (19.39)	129.96 (19.17)
Diastolic blood pressure		69.62 (12.33)	73.79 (11.92)	73.79 (11.62)
Sample size		148,540	189,552	24,183

**Fig 1:**
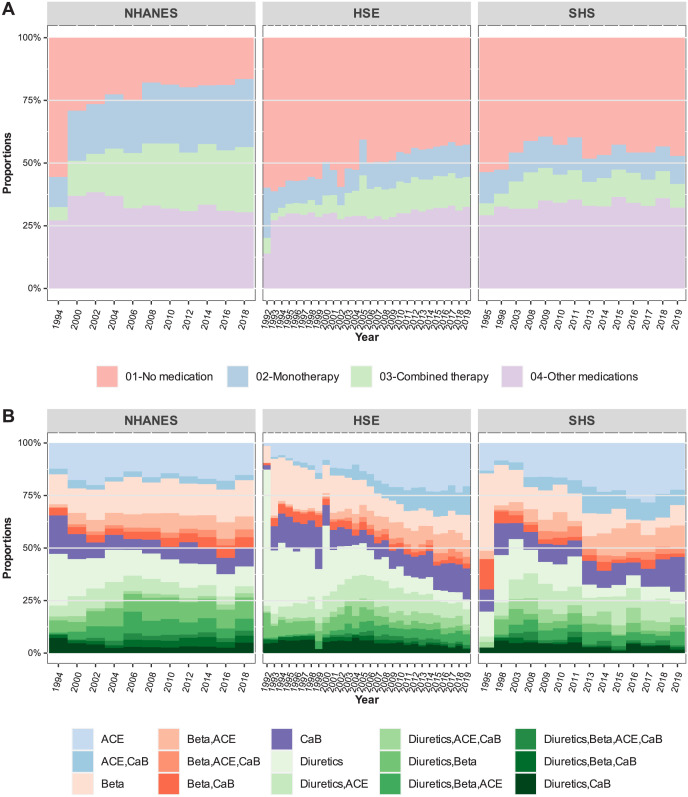
**A** Medication intake in survey respondents. **B** Antihypertensive medication regimens in respondents under antihypertension therapy over time.

As illustrated in Fig 1, countries differ regarding the proportion of respondents taking prescription medication and the specific type of antihypertensive treatment regime. The proportion of individuals in the NHANES study taking any prescription medication or being under antihypertensive treatment has increased since 1994. By the end of 2018, approximately 50% of the NHANES respondents were under some form of antihypertensive drug treatment (see Panel A, Fig 1, and the additional tables in the S1 Appendix). The results for the HSE and SHS samples also suggest an increase over time in the proportion of respondents taking antihypertensive medication; however, this increase has been less pronounced than in the NHANES samples. By the year 2019, about 25% of respondents in HSE and 20% in SHS were taking at least one antihypertensive medication (i.e., monotherapy or combined therapy), which is a larger proportion in comparison to the survey results in 1995, in which the proportions were 13% and 17%, respectively.

A more detailed analysis of the time series of the four major drug classes and their combinations reveals country-specific differences regarding the particular blood-pressure lowering regimes most frequently prescribed in each country (Panel B, Fig 1). The prescription of beta blockers and their combinations with ACE inhibitors or calcium-channel blockers has increased in NHANES between 1994 and 2018. On the contrary, in the HSE and SHS samples, the use of ACE inhibitors and their combination with calcium-channel blockers has largely increased during the entire observation period, whereas blood pressure lowering regimens based on diuretics have become less frequent over time, especially in the HSE samples (Panel B, Fig 1).

**Fig 2:**
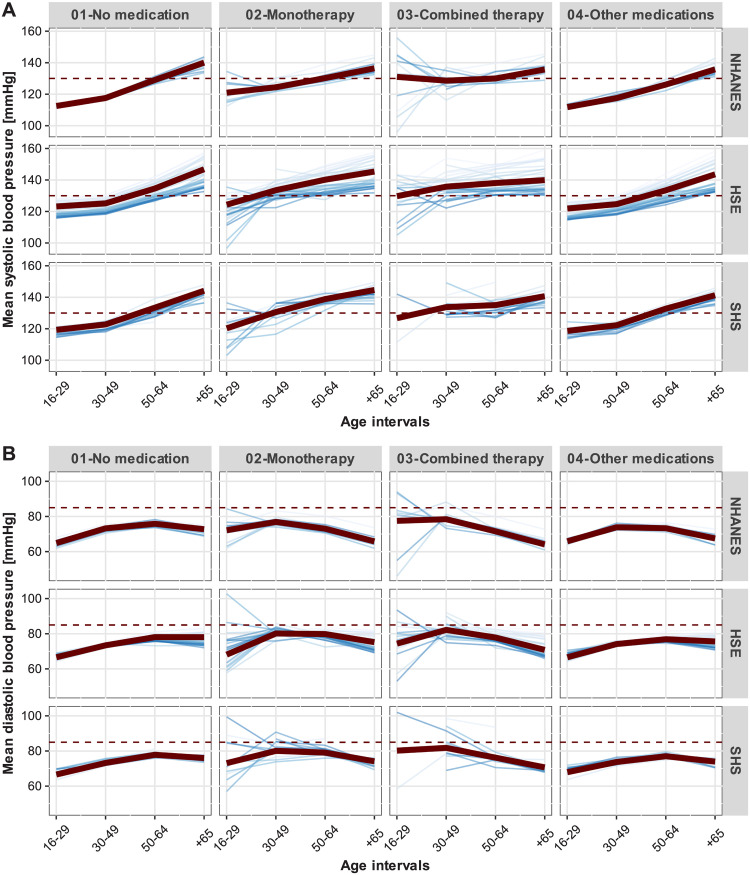
Average blood pressure measurements by use of antihypertensive medications and respondents’ age. Thick line in the foreground: grand average over all survey years. Thin lines in the background: average values for each survey year with darker colours indicating more recent surveys. **A** Systolic blood pressure. **B** Diastolic blood pressure. Thick line in the foreground: grand average over all survey years. Thin lines in the background: average values in each survey year, with darker colours indicating more recent surveys. The dotted horizontal lines represent the lower cut-off points of high normal systolic and diastolic BP grading in the European guidelines (2018): 130 and 85 mmHg, respectively [7].

Concerning the average systolic BP over the life course of respondents in each country, Panel A of Fig 2 illustrates the well-known monotonic increase of average systolic BP with age. However, because individuals taking antihypertensive medication have consistently higher systolic BP during their life span—especially persons in the combined therapy category—the growth rates of systolic BP with advancing age among those individuals tend to be lower in comparison to individuals who either take other medications or do not take any medication. Despite some country-specific patterns, by age 40–50, the average systolic BP among respondents taking antihypertensive medication is higher than the 130 mmHg cut-off point of high-normal BP. As a matter of fact, it can be observed that, at the population level, systolic BP after age 65 will be higher than 130 mmHg, irrespective of whether respondents do not take any medication or take antihypertensives or other medications. On the other hand, the diastolic BP measurements depicted in Panel B of Fig 2 are characterised by the well-known non-linear association between age and average diastolic BP, with younger and older individuals having lower diastolic BP measurements than middle-aged respondents. In contrast to the systolic BP measurements, there is a tendency for lower diastolic BP among individuals under antihypertensive drug monotherapy or combined therapy in comparison to the values of individuals receiving no medication or taking other medications. Furthermore, the results suggest that in all samples and countries, average diastolic BP remains under the 85 mmHg cut-off value for diagnosing high-normal diastolic BP.

**Fig 3:**
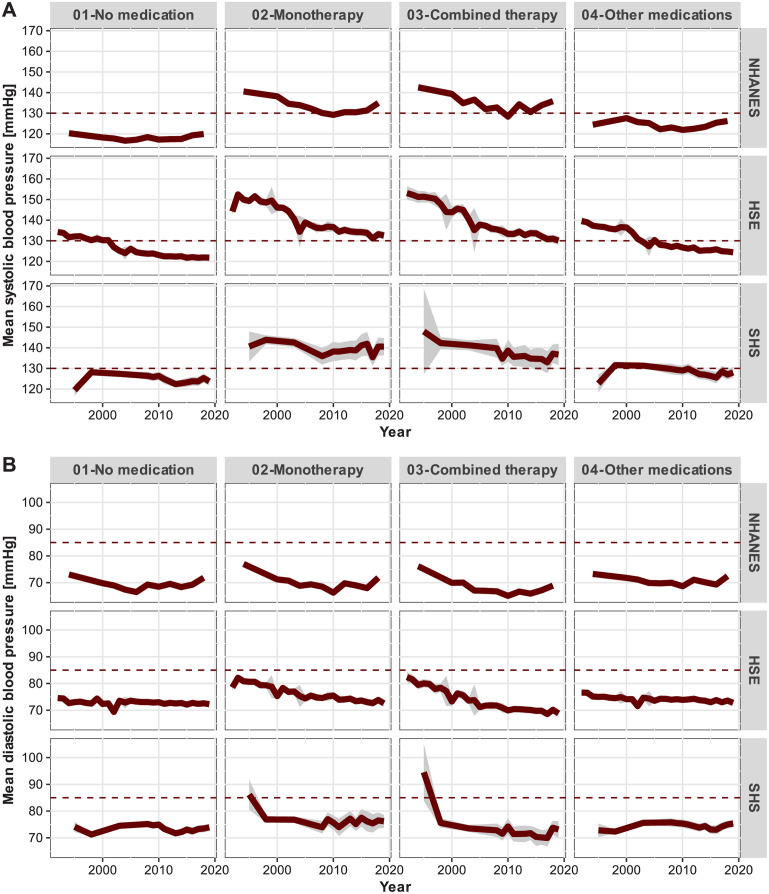
Average blood pressure measurements and 95% confidence stripes by use of antihypertensive medications and survey year. **A** Systolic blood pressure. **B** Diastolic blood pressure. The dotted horizontal lines represent the lower cut-off points of high normal systolic and diastolic BP grading in the European guidelines (2018): 130 and 85 mmHg, respectively [7].

From the perspective of the time series of average BP measurements over all age classes, the visual inspection of Panel A of Fig 3 suggests that average systolic BP has been much higher in the HSE samples in comparison to NHANES and SHS. Moreover, there has been a more pronounced decrease in average systolic BP in HSE among respondents taking antihypertensive medications. In contrast, average systolic BP among individuals who either do not take any medication or other medications has remained rather constant over time in both NHANES and SHS. However, systolic BP among NHANES respondents under antihypertensive drug treatment may have been decreasing in the period 1990–2010 and increasing in the most recent surveys in 2012–2018. In the SHS samples, on the contrary, the time trends of average systolic BP among those taking antihypertensive drugs are much flatter and do not point to substantial reductions of systolic BP at the population level. For all countries and samples considered, systolic BP among respondents taking antihypertensive medications has remained over the 130 mmHg cut-off point of high-normal BP. In contrast, the average systolic BP of those who either do not take any medications or take other medications has usually remained in the normal systolic BP band between 120–129 mmHg. Concerning average diastolic BP, the results depicted in Panel B of Fig 3 show somewhat less variation within and across samples over time. Nonetheless, the time series of average diastolic BP suggests that there may have been a stronger decline in diastolic BP among respondents in the combined therapy category, especially in the HSE and SHE samples. Moreover, this decline seems to have resulted in even lower diastolic BP levels in comparison to average diastolic BP among individuals in the other medication categories. Noteworthy is the observation that diastolic BP at the population level usually lies in the 70–80 mmHg interval and, hence, in the optimal or normal diastolic BP band (<80 mmHg).

### Regression analysis

Concerning the results of the regression analyses, the effect size estimates of the fully adjusted models confirm the observation that the systolic BP measurements in the samples are consistently higher among individuals taking antihypertensive drugs in comparison to persons who do not take any medication (Table 2). However, given the fact that there has been a time trend of decreasing systolic BP in the single surveys and the pooled dataset as well, the systolic BP reductions among those taking antihypertensive drugs has been more pronounced in comparison to persons not taking medications, especially in the HSE samples. The effect size estimates of the antihypertensive drugs and time interactions in the pooled dataset suggest that the average decrease of systolic BP over time has been similar among individuals under antihypertensive monotherapy or combined therapy (−0.28 99% CI [−0.30; −0.25] and −0.27 99% CI [−0.30; −0.24] mmHg per year, respectively). The analysis of pooled data also reveals that the average increase in systolic BP with age is substantial and amounts to 20.89 99% CI [20.65; 21.14] mmHg by age 65 and older. Age is thus the most important confounding factor accounting for most systolic BP variance between medication categories (see S1 Appendix for the results of the unadjusted models). However, there are some country-specific associations between individual characteristics and systolic BP measurements. Whereas systolic BP differences between men and women are small in NHANES in comparison to the HSE and SHS samples, the age-dependent systolic BP growth rates among individuals aged between 20 and 64 are larger in NHANES than in HSE and SHS. Moreover, the effect size estimates of smoking and BMI are larger in the HSE and SHS samples in comparison to NHANES.

**Table 2:** Linear mixed regression models of systolic blood pressure. Regression coefficients *β* and 99% confidence intervals. *m* and *n*: number of random-effect strata levels and sample size, respectively. *σ*: standard deviation of the random intercepts and residuals.

Variable	NHANES *β* 99% CI	HSE *β* 99% CI	SHS *β* 99% CI	Pooled *β* 99% CI
Intercept	114.18 [113.24; 115.11]	125.51 [124.90; 126.12]	119.37 [117.74; 121.00]	120.67 [119.93; 121.40]
**Antihypertensive medications and time. Main effects and interactions**				
Monotherapy (ref. no medications)	7.15 [6.32; 7.98]	6.37 [5.83; 6.91]	4.57 [2.36; 6.77]	6.76 [6.31; 7.20]
Combined therapy	6.09 [5.16; 7.02]	5.44 [4.70; 6.19]	2.78 [0.22; 5.33]	5.60 [5.04; 6.17]
Other medications	1.22 [0.53; 1.91]	1.20 [0.83; 1.57]	−1.35 [−2.79; 0.09]	1.18 [0.85; 1.50]
Monotherapy × Year	−0.25 [−0.30; −0.21]	−0.35 [−0.39; −0.31]	−0.16 [−0.29; −0.03]	−0.28 [−0.30; −0.25]
Combined therapy × Year	−0.21 [−0.25; −0.16]	-0.47 [−0.52; −0.43]	-0.31 [−0.45; −0.17]	−0.27 [−0.30; −0.24]
Other medications × Year	−0.10 [−0.13; −0.06]	-0.14 [−0.17; −0.11]	0.02 [−0.07; 0.10]	−0.11 [−0.13; −0.09]
Year	−0.14 [−0.19; −0.10]	−0.62 [−0.64; −0.60]	−0.30 [−0.38; −0.23]	−0.58 [−0.60; −0.56]
**Individual characteristics**				
Male (ref. female)	0.15 [−0.05; 0.34]	4.56 [4.37; 4.75]	5.10 [4.53; 5.67]	2.32 [2.19; 2.46]
30–49 years (ref. 16–29 years)	6.01 [5.67; 6.34]	1.79 [1.50; 2.07]	2.35 [1.42; 3.29]	3.61 [3.40; 3.83]
50–64 years	14.32 [13.96; 14.67]	11.61 [11.29; 11.93]	13.20 [12.20; 14.19]	12.99 [12.76; 13.23]
65 years and older	21.49 [21.13; 21.85]	20.59 [20.25; 20.94]	21.38 [20.28; 22.48]	20.89 [20.65; 21.14]
Smoking (ref. no)	−0.15 [−0.40; 0.11]	0.80 [0.59; 1.01]	0.84 [0.24; 1.44]	0.24 [0.08; 0.40]
BMI 26–30 (ref. 10–25)	1.01 [0.75; 1.27]	4.25 [4.03; 4.47]	4.16 [3.49; 4.83]	2.94 [2.78; 3.11]
BMI 31–35	1.40 [1.11; 1.68]	7.59 [7.29; 7.88]	6.76 [5.93; 7.58]	4.49 [4.29; 4.69]
BMI +35	2.83 [2.53; 3.13]	9.54 [9.11; 9.96]	8.26 [7.13; 9.39]	5.85 [5.62; 6.08]
**Surveys**				
HSE (ref. NHANES)				5.35 [3.82; 6.89]
SHS				5.71 [3.85; 7.57]
*m*	148	32	21	201
*n*	136,056	116,931	13,467	266,454
*σ*_*strata*_	2.71	1.4	1.04	3.97
*σ*_*residual*_	18.05	16.45	16.55	17.4

The results of the regression analysis corresponding to the diastolic BP measurements indicate that persons who take antihypertensive medications usually have higher diastolic BP levels in comparison to those who do not take any medication, albeit the diastolic BP differences are smaller than the systolic BP ones (Table 3). Although the analysis of pooled data suggests a general trend of decreasing diastolic BP values, this decrease has been more pronounced in the HSE and SHS samples than in NHANES. In addition, the results of the pooled analysis suggest that the average decrease in diastolic BP per year has been somewhat larger among persons under combined therapy, rather than monotherapy (−0.34 99% CI [−0.36; −0.32] and −0.31 99% CI [−0.32; −0.29] mmHg per year, respectively). In contrast to the results obtained in the systolic BP regression models, the diastolic BP levels of males are consistently higher in all surveys. Moreover, the age-dependent non-linear growth rates of diastolic BP measurements seem to show some country-specific patterns, with NHANES being associated with the largest diastolic BP decreases for persons aged 65 and older. The effect size estimates of smoking behaviour and BMI are larger in HSE and SHS than in the NHANES samples. Finally, in both the systolic and diastolic BP regression models, average BP measurements are substantially higher in the HSE and SHS than the NHANES samples (Tables 2 and 3).

**Table 3:** Linear mixed regression models of diastolic blood pressure. Regression coefficients *β* and 99% confidence intervals. *m* and *n*: number of random-effect strata levels and sample size, respectively. *σ*: standard deviation of the random intercepts and residuals.

Variable	NHANES *β* 99% CI	HSE *β* 99% CI	SHS *β* 99% CI	Pooled *β* 99% CI
Intercept	69.87 [69.12; 70.63]	65.25 [64.89; 65.62]	64.09 [63.03; 65.16]	63.15 [62.75; 63.56]
**Antihypertensive medications and time. Main effects and interactions**				
Monotherapy (ref. no medications)	2.35 [1.83; 2.88]	3.91 [3.55; 4.27]	2.57 [1.12; 4.02]	4.60 [4.31; 4.89]
Combined therapy	1.66 [1.07; 2.25]	2.76 [2.26; 3.26]	0.26 [−1.42; 1.94]	3.01 [2.64; 3.38]
Other medications	1.31 [0.87; 1.75]	0.75 [0.50; 0.99]	−0.77 [−1.72; 0.18]	1.55 [1.34; 1.76]
Monotherapy × Year	−0.11 [−0.14; −0.08]	−0.32 [−0.35; −0.30]	−0.14 [−0.23; −-0.06]	−0.31 [−0.32; −0.29]
Combined therapy × Year	−0.15 [−0.18; −0.12]	−0.46 [−0.49; −0.43]	−0.28 [−0.37; −0.19]	−0.34 [−0.36; −0.32]
Other medications × Year	−0.03 [−0.05; −0.00]	−0.07 [−0.09; −0.06]	0.02 [−0.04; 0.07]	−0.08 [−0.09; −0.07]
Year	−0.39 [−0.43; −0.36]	−0.10 [−0.11; −0.09]	0.02 [−0.03; 0.07]	−0.11 [−0.12; −0.10]
**Individual characteristics**				
Male (ref. female)	2.25 [2.13; 2.38]	2.86 [2.73; 2.99]	2.17 [1.80; 2.55]	2.60 [2.51; 2.68]
30–49 years (ref. 16–29 years)	8.95 [8.73; 9.16]	6.51 [6.32; 6.70]	5.71 [5.09; 6.32]	7.33 [7.19; 7.47]
50–64 years	8.07 [7.84; 8.29]	10.66 [10.45; 10.88]	9.53 [8.87; 10.19]	9.44 [9.29; 9.59]
65 years and older	1.12 [0.89; 1.35]	8.65 [8.42; 8.88]	6.02 [5.29; 6.75]	4.47 [4.32; 4.63]
Smoking (ref. no)	−0.10 [−0.25; 0.06]	0.30 [0.16; 0.44]	0.58 [0.19; 0.98]	0.14 [0.04; 0.24]
BMI 26–30 (ref. 10–25)	0.59 [0.43; 0.75]	2.88 [2.73; 3.03]	2.96 [2.52; 3.40]	2.01 [1.90; 2.12]
BMI 31–35	1.42 [1.24; 1.59]	5.15 [4.95; 5.34]	5.20 [4.66; 5.75]	3.44 [3.31; 3.57]
BMI +35	1.68 [1.49; 1.87]	6.38 [6.10; 6.66]	7.57 [6.83; 8.32]	3.95 [3.80; 4.10]
**Surveys**				
HSE (ref. NHANES)				3.70 [2.90; 4.49]
SHS				3.92 [2.96; 4.89]
*m*	148	32	21	201
*n*	136,056	116,931	13,467	266,454
*σ*_*strata*_	3.03	0.78	0.67	2.04
*σ*_*residual*_	11.36	10.99	10.9	11.35

**Fig 4:**
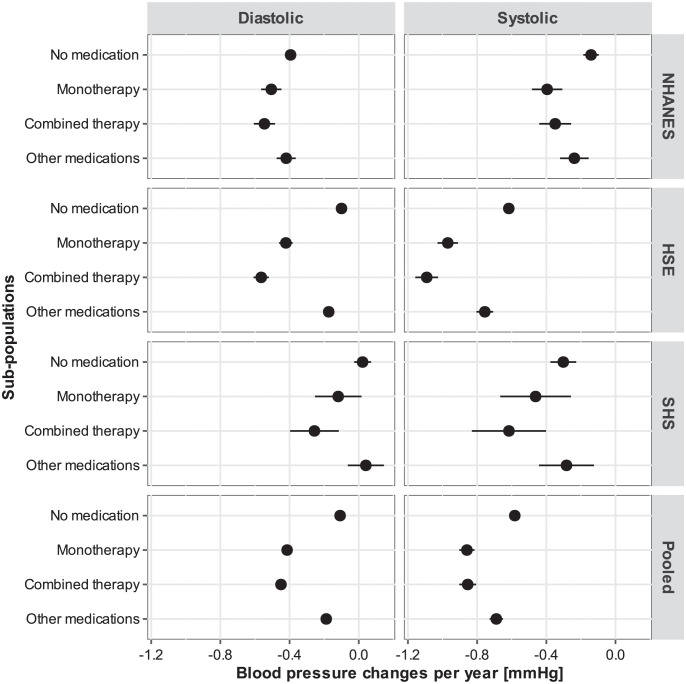
Forest plots of the interaction effects (*β*_2_) and confidence intervals reported in Tables 2 and 3. The reference category is “no medication”, which represents the secular trends of the sub-population that is not under antihypertensive treatment.

The regression coefficients of year, antihypertensive medication, and time interactions reported in the pooled analyses (Tables 2 and 3) indicate that the secular trends of BP reduction have been substantial. For the whole period 1992–2019, systolic and diastolic BP decreased by −16.24 99% CI [−16.80; −15.68] and −3.08 99% CI [−3.36; −2.80] mmHg, respectively. On the other hand, in comparison to individuals who are not taking any medication, the net potential public health impact of the use of hypertensive medications on systolic BP between 1992–2019 among individuals in antihypertensive monotherapy and combined therapy amounted to −8.56 99% CI [−8.34; −8.77] and −8.68 99% CI [−8.33; −9.03] mmHg (Table 2), whereas the average reduction of diastolic BP in the same period corresponded to −5.56 99% CI [−5.71; −5.42] and −6.55 99% CI [−6.78; −6.32] mmHg (Table 3), respectively. A summary of the main results of the interaction effects is provided graphically in the forest plots (Fig 4).

## Discussion

The results of the present study suggest that the changes in the average BP levels over time at the population level are more strongly related to secular trends, rather than changes in the proportion of individuals taking antihypertensive medications. On the one hand, the temporal patterns of decreasing or increasing BP measurements seem to affect all sub-populations, i.e., individuals taking no medications as well as those taking antihypertensive or other medications. Despite the fact that antihypertensive medications have been found to be effective in reducing BP (e.g., reduction of about 4.6/2.1 mmHg in systolic and diastolic BP for ACE inhibitors, respectively [1]), the present results suggest that there have been secular trends in systolic BP that have exerted a major decrease in BP measurements, especially in the English and Scottish samples. As reported in the results section, the potential public health impact of antihypertensive medications on systolic and diastolic BP for combined therapy amounted to −8.68 99% CI [−8.33; −9.03] mmHg and −6.55 99% CI [−6.78; −6.32] mmHg. In contrast, the secular trends amounted to −16.24 99% CI [−16.80; −15.68] and −3.08 99% CI [−3.36; −2.80] mmHg, for systolic and diastolic BP, respectively. At the same time, given the persistence of substantially higher levels of BP measurements among persons taking antihypertensive medications, especially systolic BP, the secular trend of decreasing BP has been steeper in this sub-population (Tables 2 and 3). On the other hand, the results suggest that the increasing proportion of individuals taking antihypertensive medication may have had a relatively smaller effect on the distribution of BP measurements, as the average decline of BP in all studies across sub-populations has been similar yet somewhat more pronounced in England (Fig 3). The present results agree well with the findings reported by the NCD-RisC study [17], namely, that the changes in the average BP levels from 1985 to 2016 has been largely driven by secular trends, despite the fact that the NCD-RisC analyses were not based on detailed information about antihypertensive medications. The present findings expand the scope of the NCD-RisC study by providing more accurate estimates of the differential contribution of non-pharmaceutical factors and antihypertensive medications to the changes in the population distribution of BP measurements by taking into account the major antihypertensive medication classes and the changing distribution of BMI values and demographic structure in three countries.

Furthermore, the analyses suggest that the potential effects of antihypertensive medications on BP measurements are different for systolic and diastolic BP depending on whether respondents are under monotherapy or combined antihypertensive therapy. Whereas the effect sizes of monotherapy and combined therapy in reducing systolic BP per year tend to be of similar magnitude (−0.28 99% CI [−0.30; −0.25] and −0.27 99% CI [−0.30; −0.24], respectively), the annual reduction of diastolic BP among persons under combined therapy tends to be slightly larger than among those in monotherapy (e.g., −0.34 99% CI [−0.36; −0.32] and −0.31 99% CI [−0.32; −0.29], respectively). The decline in diastolic BP among those under antihypertensive treatment in the whole observation period 1992–2019 has been even steeper than among those without medications (−6.55 99% CI [−6.78; −6.32] vs. −3.08 99% CI [−3.36; −2.80] mmHg). This observation agrees with previous research findings suggesting that the treatment of isolated systolic hypertension, i.e., the leading diagnostic criterion for initiation of antihypertensive drug therapy especially in the elderly population, is concomitant with a decrease in diastolic BP [28]. Given the fact that systolic BP targets are more difficult to achieve [29], the intensification of antihypertensive therapy or targeting lower systolic BP thresholds, e.g., <130 mmHg [30], will be associated with a larger decrease in diastolic BP, as suggested by the present and previous findings [28, 31, 32]. From a population health perspective, the time trends by age and survey year suggest the existence of two systolic BP sub-populations: a sub-population characterised by predominantly lower systolic average BP measurements (120–130 mmHg) and a sub-population of individuals with persistently higher systolic average BP measurements (130–150 mmHg) who are under antihypertensive drug therapy. In both systolic BP sub-populations, however, the age effects are highly consistent across surveys and will ultimately be associated with an average systolic BP above 130 mmHg at about age 65 and older.

When interpreting the results, readers should keep in mind that this is not a clinical study, as the units of observation are the populations in the countries considered in the analyses. In this respect, the present study does not focus on the impact of specific treatment recommendations for single individuals, medication combination algorithms, dosage, intake frequency, nor the differences between the American (ACC/AHA) and European (ESC/ESH) guideline recommendations [7, 30]. For instance, the indication of beta-blockers is usually recommended for persons presenting co-morbidities such as angina, post-myocardial infarction, or heart failure, whereas specific BP targets vary depending on multiple diagnostic criteria including age, presence of chronic kidney disease, diabetes, or stable ischaemic heart disease [7, 30]. Moreover, the American and European guidelines differ as to the BP targets, the initiation of therapy, the addition of further medications, and the particular cardiovascular risk assessment algorithms recommended [6]. At the same time, physicians’ prescription and treatment choices have been found to differ from the guideline recommendations and to depend on physicians’ characteristics (e.g., cardiologists vs. general practitioners) or familiarity with the guidelines [33, 34].

Notwithstanding these differences in diagnosis, treatment, and BP targets between the hypertension treatment guidelines, the present analyses showed a remarkable convergence of association patterns across surveys. This can be observed from the fact that the estimates of the regression models are of similar magnitude in all samples (e.g., Table 2). In addition, the largest decrease in average systolic BP was observed in the English samples (−0.62 99% CI [−0.64; −0.60]) and not in NHANES (−0.14 99% CI [−0.19; −0.10]), despite the tendency of the American guidelines to define lower BP targets than in Europe and, consequently, to treat a larger number of individuals [6, 30]. In fact, as the pooled regression analyses have taken explicitly into account the country-specific BP levels in the respective sub-populations and the sampling variance in the random effects specification in each survey year, the estimates reported here are robust marginal effect size estimates of the potential public health impact of antihypertensive therapy at the population level.

On the other hand, the benefits of the intensification of antihypertensive drug therapy (e.g., lower BP targets and additional medications) have been found to be outweighed by the harms associated with that intervention [35, 36]. The present results therefore emphasise the tremendous impact lifestyle modifications may still have on lowering BP levels on the public health scale, especially the systolic component, without incurring additional risks of adverse reactions (e.g., [37]). For instance, there is strong evidence that endurance, dynamic resistance, and isometric resistance training are as effective in reducing both systolic and diastolic BP as antihypertensive medication, with average effect sizes of about 5–11 mmHg [38, 39]. Similarly, there is also strong evidence that weight loss, the so-called dietary approaches to stop hypertension (DASH), and sodium reduction diets, can also have a substantial benefit, especially among hypertensive individuals, with average systolic and diastolic BP reductions amounting to about 4.4/3.5 mmHg, 10/5 mmHg and 4.2/2 mmHg, respectively [40]. The observation that diastolic BP among individuals receiving the combined therapy has decreased at a higher rate than among individuals taking no antihypertensive medications (−6.51 99% CI [−7.44; −5.58] vs. −3.08 99% CI [−3.36; −2.80]) agrees well with previous reports of increased risk of hypotension associated with the intensification of antihypertensive therapy [28, 41, 42]. The importance of improving the efficacy and safety of systolic BP control can be easily observed by noting that, according to the cost-benefit analysis of Ogden and colleagues, one cardiovascular disease death could be prevented by achieving a 12 mmHg reduction in systolic BP in 8 persons with high cardiovascular risk (i.e., the number-needed-to-treat) [43]. Moreover, even though the public health impact of antihypertensive medication use seems to be larger among respondents with higher BP measurements, there are some signs of stagnating or even increasing systolic BP measurements among this sub-population despite increasing uptake of antihypertensive medication, especially in the U.S. (Fig 3 and Table 2). In fact, the more pronounced non-linear trajectory of diastolic BP in the NHANES could be an indication of a higher prevalence of aortic wall stiffening and atherosclerosis in the U.S. elderly population [44, 45]. Hence, more efforts seem to be warranted in the development of effective public health interventions focusing on modifiable lifestyle factors as a means to reduce BP levels throughout the lifespan, especially among the sub-population of individuals with persistently high systolic BP.

### Strengths and limitations

A major strength of the present investigation is the fact that the analyses are based on large representative samples of the general population. In contrast to clinical cohort studies that aim to estimate the treatment effects of antihypertensive medications in rather homogeneous and restricted samples (e.g., people with co-morbidities and very old individuals are usually excluded), the present study allows for the calculation of the potential public health impact of the use of antihypertensive medications on BP at the population level in non-clinical settings. Therefore, the usual constraints of cross-sectional data regarding the estimation of average treatment effects are less relevant for the estimation of the potential effects of the intervention on the distribution of the BP measurements at the population level, i.e., when the populations of whole countries are taken as the observational units. Moreover, the present study provides a detailed analysis of major time trends in BP measurements and antihypertensive medication use over a three-decade period in three countries and, therefore, captures a wider range of geographical variation than most clinical samples.

On the other hand, the present investigation is limited concerning a more detailed identification of the major non-pharmaceutical factors that would be able to explain to some extent the observed secular trends in decreasing BP measurements over time. In addition, long time series on intake of angiotensin-receptor blockers (ARB) were not available for most surveys, so separate analysis for this medication class could not be performed. Nonetheless, according to previous results, the magnitude of the effect sizes across different antihypertensives, including ARB, has been found to be similar [46] and, therefore, the results obtained in the present investigation are expected to be comparable to ARB as well. Furthermore, even though the present study confirms the important role of overweight as a contributor to raised BP measurements, the analyses cannot provide further information as to which illnesses, co-morbidities, dietary, and/or physical activity characteristics in each country may be more associated with raised BP and antihypertensive treatment strategies. Given that these unaccounted factors are partly related to the therapeutic approach, the estimates provided in the present study are likely overestimated and represent an upper bound of the true effects, since they are to some extent confounded by the effects of illnesses, co-morbidities, physical activity, and dietary habits. Future studies could consider the extent to which the observed treatment effects may be due to reduced cardiometabolic risk at the population level.

Even though great care was put into the harmonisation of variables within and between samples, there was some temporal variation in the data collection protocols of the surveys, especially in the HSE and SHS. However, these changes concerned only a few survey years in which some data were not collected or new coding procedures or reporting protocols were introduced. Some examples are: (i) age was reported in HSE as a continuous variable until 2013 and from 2014 on as a categorical variable; (ii) information on antihypertensive medication in SHS 2012 was not collected; or (iii) information on sampling strata in HSE and SHS slightly changed during the observation period due to re-definition of administrative areas. For the core variables corresponding to BP measurements, the largest variation between surveys was observed for the SHS samples collected in the 1990s. For the subsequent SHS survey years, however, no large deviations were observed. On the contrary, in the HSE and NHANES, the BP measurements show a high degree of consistency and reveal more distinctly the secular trends in decreasing BP (see Figs 1–3). Finally, data on the use of antihypertensives do not show signs of abrupt changes in the proportions over time (Fig 1), except for the 1994 and 1992 NHANES and HSE samples, respectively. Nonetheless, as stated in the methods section, time and survey-specific variations were taken into account in the regression models as fixed and random variation by year and sample.

Finally, even though the decline in BP measurements has been observed in several European countries, including Finland [47], Sweden [48], and Norway [49], the exact causal mechanisms accounting for these consistent secular trends in different populations are to a large extent elusive [17]. It has been speculated that changes in dietary patterns such as sodium intake could partly explain this decline [50]. At least for England, there is some evidence suggesting that the potential public health impact of national strategies to reduce salt intake between 2003–2007 amounted to −0.175 g per day/year [51]. This estimate could be related to the observed systolic BP decline in HSE of about −0.62 99% CI [−0.64; −0.60] mmHg per year (Table 2), given the fact that salt intake reductions of 2 g/day would yield a reduction of about 3.47 mmHg in systolic BP [52]. However, it is unlikely that this national strategy explains the observed BP decrease in HSE, which actually seems to have occurred already in the 1990s (Fig 3). At any rate, given the plethora of societal transformations that may affect not only the patterns of sodium intake, but also dietary habits in general (e.g., electrolytes, carbohydrates, fatty acids [53]), the frequency and intensity of physical activity, occupational exposures, smoking, or the introduction of medical interventions indirectly affecting BP [54], it is clear that more research using appropriate data is needed to substantiate potential causal mechanisms accounting for the secular trends in the distribution of BP measurements.

## Conclusions

The temporal patterns of decreasing or increasing BP measurements over time seem to affect all sub-populations, i.e., individuals taking no medications as well as those taking antihypertensive or other medications. From a public health perspective, these results point to large potential effects of non-pharmaceutical factors that may have a large impact on BP levels and that may be integrated more explicitly in the development of comprehensive and more efficient hypertension treatment options. Given the fact that the benefits of the intensification of antihypertensive drug therapy (e.g., lower BP targets and additional medications) have been found to be outweighed by the harms associated with that intervention, the present results emphasise the potential impact lifestyle modifications may have on lowering BP at the population level, especially the systolic component.

## Supporting information

S1 Appendix Supplementary file. (PDF)

S1 Checklist STROBE statement—Checklist of items that should be included in reports of observational studies. (PDF)

## Abbreviations

ACE: Angiotensin-converting enzyme inhibitors
ARB: Angiotensin-receptor blockers
ATC: Anatomical Therapeutic Chemical Classification
BP: blood pressure
ESC: European Society of Cardiology
ESH: European Society of Hypertension
NHANES: The National Health and Nutrition Examination Survey
HSE: Health Survey for England
SHS: Scottish Health Survey
